# Relationship Between Antihypertensive Medications and Cognitive Impairment: Part I. Review of Human Studies and Clinical Trials

**DOI:** 10.1007/s11906-016-0674-1

**Published:** 2016-08-05

**Authors:** Sevil Yasar, Mattan Schuchman, Jean Peters, Kaarin J. Anstey, Michelle C. Carlson, Ruth Peters

**Affiliations:** 1 Division of Geriatric Medicine and Gerontology, Department of Medicine, Baltimore, Johns Hopkins School of Medicine, Baltimore, MD 21224 USA; 2 School of Health and Related Research, University of Sheffield, Sheffield, UK; 3 Centre for Research on Ageing, Health and Wellbeing, College of Medicine, Biology and Environment Australian National University, Canberra, Australia; 4 Department of Mental Health, Johns Hopkins Bloomberg School of Public Health, Baltimore, MD 21224 USA; 5 School of Public Health, Imperial College London, London, SW7 2AZ UK

**Keywords:** Antihypertensive medication, Cognitive decline, Dementia, Alzheimer’s disease

## Abstract

**Purpose of review:**

There is an established association between hypertension and increased risk of poor cognitive performance and dementia including Alzheimer’s disease; however, associations between antihypertensive medications (AHMs) and dementia risk are less consistent. An increased interest in AHM has resulted in expanding publications; however, none of the recent reviews are comprehensive. Our extensive review includes 15 observational and randomized controlled trials (RCTs) published over the last 5 years, assessing the relationship between AHM and cognitive impairment.

**Recent findings:**

All classes of AHM showed similar result patterns in human studies with the majority of study results reporting point estimates below one and only a small number of studie*s* (*N* = 15) reporting statistically significant results in favor of a specific class.

**Summary:**

Only a small number of studies reported statistically significant results in favor of a specific class of AHM. Methodological limitations of the studies prevent definitive conclusions. Further work is now needed to evaluate the class of AHM and cognitive outcomes in future RCTs, with a particular focus on the drugs with the promising results in both animals and human observational studies.

**Electronic supplementary material:**

The online version of this article (doi:10.1007/s11906-016-0674-1) contains supplementary material, which is available to authorized users.

## Introduction

There is a long-established association between hypertension and increased risk of age-related cognitive decline and dementia [1], but the potential association between antihypertensive treatment and reduced risk of dementia has been harder to determine. The majority of observational studies, clinical trials, and systematic reviews in this area suggest that antihypertensive treatment may be associated with a decreased risk of cognitive decline and incident dementia. However, the results of individual studies vary widely; for example, one study showed a 50 % reduction in incident dementia, while another demonstrated no association between incident dementia and any type of antihypertensive use [2–5].

Attempts to further understand the discrepancies in this area have shifted attention towards the potential pleiotropic effects of the different classes of antihypertensive medication (AHM) and their potential impact on cognitive function [4, 5]. In 2009, two reviews were published on this topic. Fournier et al. reported that calcium channel blocker (CCB) and angiotensin receptor blockers (ARBs) were the most promising antihypertensive classes with regard to prevention of incident cognitive decline and dementia [5]. Shah et al. in another review favored angiotensin-converting enzyme inhibitor (ACE-I) and diuretics [4], although the numbers of constituent studies were small, two for Alzheimer’s disease (AD), four for vascular dementia, and five for any dementia outcomes [4]. Although publication in this area has expanded, none of the 16 more recent reviews (5 systematic and 11 non-systematic) provide full oversight of the newer literature. Moreover, no review to date has included a systematic update of the mechanistic animal and human studies, observational, and randomized controlled studies (RCTs) across the different classes of AHM. This review aims to provide such an update in two parts. Part 1 provides an overview of the recent human observational and clinical trial literature, and part 2 reviews the recent physiological and animal work.

## Methods

### Search Strategy

The databases Embase, PsycINFO®, Medline, Medline in process and other non-indexed citations, and PubMed were searched from 2010 to February 2016 using the search terms dementia or cognit* or mild cognitive impairment, and antihypertensives, or antihypertensive agents, or diuretic or diuretics or thiazide-like or calcium channel blocker or calcium channel blockers or calcium antagonist or angiotensin converting enzyme inhibitor or angiotensin-converting enzyme inhibitors or ACE inhibitors or angiotensin receptor blocker or angiotensin receptor blockers or ARB or beta blocker or adrenergic beta-antagonists. Where review articles were identified, reference lists were searched for original research articles published within the last 5 years.

### Inclusion and Exclusion Criteria

Included studies were required to be longitudinal, to report on cognitive decline or incident dementia, and to exclude participants with existing cognitive impairment. Studies reporting solely on change in cognitive function scores were excluded. Studies were required to include exposure to one of the antihypertensive classes of interest, CCB, ARB, ACE-I, beta blockers (BBs), and diuretics, and to have a control or comparator group.

### Article Selection

Abstracts were double read and reviewed by RP and JP. Discrepancies were resolved by discussion. Full text articles were double read by the same team and data extracted into standard tables, collated by antihypertensive class.

### Quality Assessment

Quality was assessed against the key factors given in Critical Appraisal Skills Program checklists [6] for evaluating trials and longitudinal studies and a detailed table produced. A formal scoring scheme was not used as this can lead to a loss of subtlety when assessing quality.

## Results

Searches retrieved 138 PubMed records and 522 records from Medline, PsycINFO®, and Embase. Hand searching identified two further articles. Seventeen full text articles were assessed for eligibility [3, 7–13, 14•, 15–23]. Of these, three reported solely on change in neuropsychological test score [7–9]; one had no valid control group [10]; and in one, it was not clear whether those with cognitive impairment at baseline had been excluded [11] (see Figure A in supplemental material).

The 12 articles meeting the inclusion criteria reported on 14 studies (Table A in supplemental material). One article reported the results for two randomized controlled trials, the Ongoing Telmisartin Alone and in Combination with Ramipril Global Endpoint Trial (ONTARGET), and Telmisartin Randomized Assessment Study in ACE Intolerant Subjects with Cardiovascular Disease trial (TRANSCEND) trials [3]. Five articles reported cohort studies [12, 13, 17–19], four from population-based samples [13, 17–19] and one from a clinical trial [12]. In the latter trial, the data were treated as a cohort for the purposes of the analysis since the clinical trial intervention had no impact on cognitive function [12]. Three articles reported analyses of cohorts derived retrospectively from medical record databases [16, 20, 21]; one was a systematic review but included unpublished and relevant results from two further cohort studies [14•], and two were case control analyses using records from existing medical databases [15, 22].

The reporting of baseline characteristics varied widely. However, all studies except one included both men and women, with the percentage women ranging from 2 to 3 % in the studies using the US Veteran’s Affairs data [16, 21] to close to 50 % in the cohort studies [18–21]. One study only included those with incident diabetes during the 4-year exposure period [21], and the Honolulu Asia Aging Study (HAAS) included only Japanese men who had previously participated in the Honolulu Heart Program [17]. In addition to the HAAS, five articles reported on data from the USA, the Cache County study [19], the Ginkgo Evaluation and Memory Study (GEMS) [12], and the 90+ study [14•], and two reported on data from the US Veterans Affairs medical database [16, 21]. Other studies reported on Italian [18], UK [15], French [14•], German [22], and Taiwanese [20] populations.

The clinical trials recruited participants from 40 countries around the world including sites in North and South America, Europe, Asia and Australasia, and South Africa [3]. Study size ranged from >800,000 assessed as part of the US Veterans Affairs database [16] to 204 UK 85-year olds with 3-year follow-up in the Newcastle 85+ study [13]. The majority of the studies reported mean or median follow-up between 3 and 6 years [3, 12, 13, 16, 20, 22].

All studies reported on exposure to at least one of the AHM classes under consideration, and most reported results for several classes, although the percentage exposed by class differed between studies, as described in Table B in the supplemental material.

Outcome measures and comparator groups also varied with results reported for incident AD [12, 15, 16, 19, 20], cognitive impairment [3, 13, 17, 18], vascular dementia [15], and all-cause dementia [14•, 15, 16, 21, 22]. The exposed samples were compared to those taking other antihypertensive drugs [3, 13, 14•, 15, 16, 18–20, 22], those not taking antihypertensives [3, 12, 17], or both [19, 21].

### CCBs

Six studies reported on CCBs [12, 13, 17, 19, 21, 22] (Table 1). Five reported point estimates below one in favor of CCB use reducing risk of cognitive decline or dementia [12, 13, 17, 19, 21], but only two reached statistical significance, the Newcastle 85+ study [13] and analyses of a cohort from the US Veteran’s Affairs medical database [21]. In the Newcastle 85+ study, CCB users compared to those taking other antihypertensives were less likely to experience a drop of four or more points in their Mini-Mental State Exam score. However, the relationship was borderline when a drop of more than the Reliable Change Index (RCI) was used instead [13]. The RCI is a means to calculate a measure of reliable change in a cognitive test score. In the Veteran’s Affairs data, CCB users were less likely to have a recorded diagnosis of dementia [21]. No studies reported an increased risk associated with CCB use.

**Table 1:** Human studies reporting results related to calcium channel blocker (CCB) use

Author	Study name	Adjusted results
Yasar et al. 2013 [12]	GEMS	For incident Alzheimer’s disease—drug class vs non antihypertensive users CCB HR 0.62 (0.35–1.09)
Gelber et al. 2013 [17]	HAAS	For incident cognitive impairment drug class vs non antihypertensive users CCB alone IRR 0.97 (0.75–1.27)
Peters et al. 2015 [13]	The Newcastle 85+ study	For a fall in SMMSE of ≥4 points CCB users compared to all other antihypertensive users OR 0.16 (0.03–0.79) For a fall in SMMSE greater than reliable change index (Hensel et al. 2007) CCB users compared to all other antihypertensive users OR 0.38 (0.15–1.00)
Chuang et al. 2014 [19]	Cache County study	For incident Alzheimer’s disease CCB users compared to other antihypertensive users and non-users HR 0.75 (0.55–1.04) Dihydropiridine CCB users compared to other antihypertensive users and non-users HR 0.75 (0.48–1.17) Non-dihydropiridine CCB users compared to other antihypertensive users and non-users HR 0.88 (0.60–1.30) CCB users compared to other antihypertensive users HR 0.85 (0.60–1.20) Dihydropiridine CCB users compared to other antihypertensive users HR 0.84 (0.53–1.34) Non-dihydropiridine CCB users compared to other antihypertensive users HR 0.99 (0.66–1.49)
Johnson et al. 2012 [21]	Veterans Administration cohort	For incident dementia drug class vs antihypertensive users and non-users CCB HR 0.929 (0.893–0.966)
Wagner et al. 2012 [22]	Disease Analyzer database cohort	For incident dementia, antihypertensive drug type compared to matching control CCB OR 1.10 (0.85–1.43)

### ACE-I

Seven studies reported on ACE-I use [12, 13, 15, 17–19, 22] (Table 2). Six reported point estimates below one in favor of ACE-I use reducing risk of cognitive decline or dementia [12, 13, 15, 18, 19, 22], but only two reached statistical significance, the GEMS study [12] and analyses of a cohort from the UK General Practice Research Datalink medical database (GPRD) [15]. The GEMS study reported a decreased risk of AD compared to those not using antihypertensives [12], whereas the GPRD data showed a reduced risk of probable AD, possible AD, probable vascular dementia, “unspecified or other dementia,” or “any dementia” in ACE-I users compared to other antihypertensive use [15]. None of these studies reported reductions in rates of cognitive decline. No studies reported an increased risk associated with ACE-I use.

**Table 2:** Human studies reporting results related to angiotensin-converting enzyme (ACE) inhibitor

Author	Study name	Adjusted results
Yasar et al. 2013 [12]	GEMS	For incident Alzheimer’s disease Drug class vs non antihypertensive user ACE HR 0.50 (0.29–0.83)
Gelber et al. 2013 [17]	HAAS	For incident cognitive impairment drug class vs non antihypertensive users ACE alone IRR 1.03 (0.71–1.50)
Solfrizzi et al. 2013 [18]	ILSA	For incident MCI ACE compared to other antihypertensive medications HR 0.39 (0.12–1.24) ACE compared to no antihypertensive medication HR 0.45 (0.16–1.28)
Peters et al. 2015 [13]	The Newcastle 85+ study	For a fall in SMMSE of ≥4 points ACE users compared to all other antihypertensive users OR 0.41 (0.11–1.57) For a fall in SMMSE greater than reliable change index (Hensel et al. 2007) ACE users compared to all other antihypertensive users OR 0.71 (0.28–1.79)
Chuang et al. 2014 [19]	Cache County study	Whole sample, incident Alzheimer’s disease ACE users compared to other antihypertensive users and non-users HR 0.95 (0.71–1.29) Antihypertensive medication users, incident Alzheimer’s disease ACE users compared to other antihypertensive users HR 1.06 (0.76–1.50)
Davies et al. 2014 [15]	GPRD cohort	ACE compared to other antihypertensive Probable Alzheimer’s disease OR 0.76 (0.69–0.84) Possible Alzheimer’s disease OR 0.80 (0.75–0.87) Probable vascular dementia OR 0.82 (0.75–0.91) Unspecified or other dementia OR 0.85 (0.75–0.96) Any dementia OR 0.80 (0.76–0.84)
Wagner et al. 2012 [22]	Disease Analyzer database cohort	For incident dementia Antihypertensive drug type compared to matching control ACE OR 0.84 (0.65–1.08)

### ARBs

Eight studies reported results for ARB use [3, 12, 15, 16, 20–22] (Table 3). Four reported point estimates below one in favor of ARB use reducing risk of cognitive decline or dementia [12, 15, 16, 21], and all reached statistical significance. These included GEMS (AD) [12] and three analyses of existing medical databases, two using the US Veteran’s Affairs Database [21] and reporting on AD and any dementia, and one the UK GPRD database (AD, vascular, unspecified or other dementia, or any dementia) [15]. The remaining four studies included the large multinational double-blind randomized placebo-controlled trials, ONTARGET and TRANSCEND comparing ARB use to ACE use or placebo, respectively [3], and analyses of existing medical record databases in Taiwan [20] and Germany [22]; none of which found a relationship between ARB use and mitigation of cognitive decline or risk for dementia.

**Table 3:** Human studies reporting results related to Angiotensin Receptor Blocker (ARB) use

Author	Study name	Adjusted results
Anderson et al. 2011 [3]	ONTARGET	Authors state that none of the treatment effects were changed appreciably when adjusted sequentially with key variable.
Anderson et al. 2011 [3]	TRANSCEND	
Yasar et al. 2013 [12]	GEMS	For incident Alzheimer's Disease - drug class vs non antihypertensive users ARB **HR 0.31(0.14-0.68)**
Peters et al. 2015 [13]	The Newcastle 85+ Study	For a fall in SMMSE of >=4 points: ARB users compared to all other antihypertensive users Numbers too small. For a fall in SMMSE >reliable change index (Hensel et al. 2007): ARB users compared to all other antihypertensive users Numbers too small
Li et al. 2010 [16]	Veterans Affairs cohort 2002-2006	For the outcome of Alzheimer's Disease; ARB compared to Lisinopril **HR 0.81 (0.68-0.96)** ARB compared to CV comparator **HR 0.84 (0.71-1.00)** For the outcome of dementia; ARB compared to Lisinopril **HR 0.81 (0.73-0.90)** ARB compared to CV comparator **HR 0.76 (0.69-0.84)** Plus: Those who switched from ACE to ARB during the study compared to those who remained on ACE Results given for dementia: **HR0.28 (0.24-0.32)** Combined ARB and ACE stronger results than ARB or ACE alone for both dementia and Alzheimer's Disease outcomes.
Hsu et al. 2013 [20]	Taiwan National Health Insurance cohort	For incident Alzheimer's Disease: All ARBs compared to non-ARB group **HR 1.08 (0.96-1.22).**
Johnson et al. 2012 [21]	Veterans Affairs cohort 2002-2006	For incident dementia drug class vs antihypertensive users and non-users: ARB **HR 0.763 (0.699-0.834)**
Davies et al. 2014 [15]	GPRD cohort	ARB compared to other antihypertensive Probable Alzheimer's Disease **OR 0.47 (0.37-0.58)** Possible Alzheimer's Disease **OR 0.51 (0.43-0.61)** Probable Vascular Dementia **OR 0.70 (0.57-0.85)** Unspecified or other dementia **OR 0.62 (0.47-0.81)** Any dementia **OR 0.55 (0.49-0.62)** Analysis of the association between years of defined daily dose exposure suggests a stronger relationship between longer exposure to ACE and lower OR for dementia, particularly Alzheimer’s disease. Relationship less clear for ARBs. Analysis of time lag data excluding 1,2....8 years of exposure prior to diagnosis shows stronger relationship for exposure closer to index date for ACE and probable or possible Alzheimer's Disease or probable vascular dementia. Similar but much stronger patterns seen for ARBs and probable or possible Alzheimer's Disease with ARB exposure associated with significantly lower OR for all time lag periods.
Wagner et al. 2012 [22]	Disease Analyzer Database cohort	For incident dementia: Antihypertensive drug type compared to matching control. ARB **OR 1.04 (0.66-1.64)**

### Diuretics

Eight studies reported on diuretic use [12, 13, 14•, 17, 19, 21, 22] (Table 4). Seven reported point estimates below one in favor of diuretic use reducing risk of cognitive decline or dementia [12, 13, 14•, 19, 21, 22]; three reached statistical significance, GEMS (AD) [12], the Cache County study (AD) [19], and analyses of data from the US Veteran’s Affairs database (dementia) [21]. Data from the 90+ and Three City studies, as reported in the review by Tully et al., did not reach significance [14•], and neither did analyses from the Newcastle 85+ study [13] or those using the German Disease Analyzer medical database [22]. The HAAS reported a point estimate above one without statistical significance for the outcome of cognitive impairment [17].

**Table 4:** Human studies reporting results related to diuretic use

Author	Study name	Adjusted results
Yasar et al. 2013 [12]	GEMS	For incident Alzheimer’s disease drug class vs non antihypertensive users Diuretics HR 0.51 (0.31–0.82)
Gelber et al. 2013 [17]	HAAS	For incident cognitive impairment drug class vs non antihypertensive users Diuretic alone IRR 1.01 (0.75–1.38)
Peters et al. 2015 [13]	The Newcastle 85+ study	For a fall in SMMSE of ≥4 points Thiazide and related diuretic users compared to all other antihypertensive users OR 0.70 (0.21–2.34)For a fall in SMMSE greater than reliable change index (Hensel et al. 2007) Thiazide and related diuretic users compared to all other antihypertensive users OR 0.72 (0.28–1.86)
Chuang et al. 2014 [19]	Cache County study	For incident Alzheimer’s disease Diuretic users compared to other antihypertensive users and non-users HR 0.72 (0.56–0.93) Loop diuretic users compared to other antihypertensive users and non-users HR 0.98 (0.67–1.43) Thiazide diuretic users compared to other antihypertensive users and non-users HR 0.70 (0.53–0.93) Potassium sparing users compared to other antihypertensive users and non-users HR 0.69 (0.48–0.99) For incident Alzheimer’s disease Diuretic users compared to other antihypertensive users HR 0.77 (0.56–1.06) Loop diuretic users compared to other antihypertensive users HR 1.09 (0.73–1.64) Thiazide diuretic users compared to other antihypertensive users HR 0.75 (0.54–1.04) Potassium sparing users compared to other antihypertensive users HR 0.74 (0.50–1.10)
Johnson et al. 2012 [21]	Veterans Affairs cohort 2002–2006	For incident dementia drug class vs antihypertensive users and non users Diuretics HR 0.864 (0.826–0.904)
Tully et al. 2016 [14•]	Meta-analysis reporting unpublished data from the 90+ study	For incident dementia Diuretic use compared to control HR 0.90 (0.73–1.11)
Tully et al. 2016 [14•]	Meta-analysis reporting unpublished data from the Three Cities study	For incident dementia Diuretic use compared to control HR 0.78 (0.38–1.59)
Wagner et al. 2012 [22]	Disease Analyzer database cohort	For incident dementia, antihypertensive drug type compared to matching control Diuretics OR 0.89 (0.67–1.19)

### BBs

Six studies reported on BB use [12, 13, 17, 19, 21, 22] (Table 5). Four reported point estimates in favor of BB use reducing risk of cognitive impairment (HAAS) [17], AD (GEMS) [12], and dementia (analyses from the US Veteran’s Affairs and German Disease Analyzer medical databases) [12, 22], respectively. In the Cache County study, point estimates were below one for comparisons of BB users to other antihypertensive users and non-users combined but above one for comparisons to other antihypertensive users alone. Neither comparison was statistically significant [19]. In the Newcastle 85+ study, the point estimate was also above one, suggesting the potential for increased risk but without statistical significance [13].

**Table 5:** Human studies reporting results related to beta blocker (BB) use

Author	Study name	Adjusted results
Yasar et al. 2013 [12]	GEMS	For incident Alzheimer’s disease—drug class vs non antihypertensive users BB HR 0.58 (0.36–0.93)
Gelber et al. 2013 [17]	HAAS	For incident cognitive impairment drug class vs non antihypertensive users BB alone IRR 0.66 (0.48–0.94)
Peters et al. 2015 [13]	The Newcastle 85+ study	For a fall in SMMSE of ≥4 points BB users compared to all other antihypertensive users OR 2.60 (0.96–7.07) For a fall in SMMSE greater than reliable change index (Hensel et al. 2007) BB users compared to all other antihypertensive users OR 1.68 (0.74–3.83)
Chuang et al. 2014 [19]	Cache County study	For incident Alzheimer’s disease BB users compared to other antihypertensive users and non-users HR 0.90 (0.67–1.21) BB users compared to other antihypertensive users HR 1.02 (0.73–1.43)
Johnson et al. 2012 [21]	Veterans Affairs cohort 2002–2006	For incident dementia, drug class vs antihypertensive users and non-users BB HR 0.956 (0.918–0.995)
Wagner et al. 2012 [22]	Disease Analyzer database cohort	For incident dementia, antihypertensive drug type compared to matching control BB OR 0.79 (0.61–0.99)

Given the diversity of study designs, comparator groups, and outcomes, it was not possible to meaningfully pool any results.

### Quality Assessment, Human Observational, Cohort Studies, and Trials

Multiple sources of bias are contained within the eligible studies. These include the differing designs, populations, comparator groups, calculation of exposure to the antihypertensive classes of interest, evaluation of outcome measures, and differing lengths of follow-up. See Tables C1–3 for details.

## Discussion

The importance of dementia as a clinical and public health issue is rapidly increasing as the population ages [23]. Thus, identifying new and effective approaches to prevention or treatment is critical. Due to the lengthy process of developing new medications, there has been a recent surge in interest towards re-purposing currently available medications for the treatment of AD, including AHM. In this paper, we provide an extensive review of 15 observational and randomized controlled studies published over the last 5 years, assessing the relationship between AHM and cognitive impairment.

Previous studies have shown a possible protective effect of certain AHM against AD risk [1], and it has been suggested that this protective effect is independent of, or in addition to, the blood pressure-lowering effect [4, 5].

Six observational studies reported on CCB use as a group and risk of cognitive decline or impairment [12, 13, 17, 19, 21, 22], but only two have reached statistical significance [13, 21]. However, no recent observational study has evaluated any specific medications. This is most likely due to lack of power, raising the need for either larger studies or meta-analysis of multiple observational studies to provide information and guidance for future RCTs.

Seven observational studies reported on ACE-I use [12, 13, 15, 17–19, 22], but only two studies reached statistical significance [12, 15]. Again, no observational study has evaluated any specific medication, most likely due to lack of power. The large multinational double-blind randomized placebo-controlled trial ONTARGET compared the ACE-I ramipril to the ARB telmisartan and found no significant difference [3]. There was also no significant difference in a further large multinational double-blind randomized placebo-controlled trial, TRANSCEND, when comparing ARB (telmisartan) use to placebo [3]. Overall, eight observational studies reported results for ARB use as a group [3, 12, 15, 16, 20–22], of which four have reported risk reduction of cognitive decline or dementia [12, 15, 16, 21].

There are eight observational studies reporting on diuretic use [12, 13, 14•, 17, 19, 21, 22], with three reporting significant risk reduction of cognitive decline or dementia [12, 19, 21]. Six observational studies reported on BB use [12, 13, 17, 19, 21, 22], with four studies reporting risk reduction of cognitive impairment [12, 17, 21, 22], respectively.

Previous animal studies and also RCTs with AHM have shown that blood pressure reduction, particularly in close proximity to development of cognitive impairment, does not alter dementia risk. Thus, other mechanisms involved in AD or dementia development should to be explored; however, medications used in mechanistic studies have been different agents to those used so far in RCTs.

## Conclusions

All classes of AHM show similar patterns of results. The majority of study results report point estimates below one, and a small number of studies report statistically significant results in favor of a specific class. The data is most mixed with regard to ARBs, where four out of eight studies report significantly in favor of ARBs and four, including two double-blind randomized controlled trials, report null results.

Inconsistencies in the sources of evidence, the use of human populations of differing ages, sex ratios, and prior or concurrent exposure to AHM limit the possibility of drawing firmer conclusions. Differing treatment times in studies, methods for calculating exposure to treatment, outcome measures, and study designs with some studies potentially including assessments during early, but as yet undiagnosed, dementia also weaken the ability to draw conclusions. The relative lack of information on blood pressure levels and use of differing covariates further serves to complicate interpretations. These limitations restrict our ability to draw wider ranging conclusions about use of specific antihypertensive classes, subclasses, or individual drugs. Further work needs to include cognitive outcomes such as early decline and select robust covariates and comparator groups. In addition, future work should explore key subgroups such as those with prior stroke and cardiovascular disease, who are at higher risk for developing dementia. AHM that have had promising results in animals and larger human observational studies are the specific agents that should be selected for future RCTs.

## Electronic supplementary material

Below is the link to the electronic supplementary material.

ESM 1, Figure S1 Study selection, human observational, cohort studies and trials (DOCX 28.1 kb)

ESM 2 (DOCX 17.6 kb)

ESM 3 (DOCX 21.7 kb)

ESM 4 (DOCX 29.7 kb)
