# Supplementary material for: Relationship Between Antihypertensive Medications and Cognitive Impairment: Part I. Review of Human Studies and Clinical Trials
Source: Curr Hypertens Rep. 2016 Aug 5;18:67. doi: 10.1007/s11906-016-0674-1 (PMC4975763; doi:10.1007/s11906-016-0674-1)
Supplement: Supplementary file 1 — Study selection, human observational, cohort studies and trials (DOCX 28.1 kb) [file 11906_2016_674_MOESM1_ESM.docx]

Figure A. Study selection, human observational, cohort studies and trials.

Records identified through database searching
n =522 (Medline, PsycINFO®, Embase)

n=138 (PubMed)

n =522 (Medline, PsycINFO®, Embase) n=138 (PubMed)

Full-text articles assessed for eligibility
(n =17)

Full-text articles excluded, with reasons
(n = 3 Outcome of neuropsychological change only)

(n=1 No equivalent comparator group)

(n=1 not clear that those with cognitive impairment at baseline were excluded)

Studies included in qualitative synthesis
(n =12 articles, 14studies/trials)

Studies included in quantitative synthesis 0

Additional records identified through reference lists and other sources n =2

Records screened
