# Supplementary material for: Relationship Between Antihypertensive Medications and Cognitive Impairment: Part I. Review of Human Studies and Clinical Trials
Source: Curr Hypertens Rep. 2016 Aug 5;18:67. doi: 10.1007/s11906-016-0674-1 (PMC4975763; doi:10.1007/s11906-016-0674-1)
Supplement: Supplementary file 2 — (DOCX 17.6 kb) [file 11906_2016_674_MOESM2_ESM.docx]

Table A. Eligible human studies

| Author | Study name | Design |
| --- | --- | --- |
| Anderson et al 2011 | Data from the Ongoing Telmisartin Alone and in Combination with Ramipril Global Endpoint Trial (ONTARGET) | Double blind randomised trial with active control group |
| Anderson et al 2011 | Data from the Telmisartin Randomised Assessment Study in ACE Intolerant Subjects with Cardiovascular Disease trial (TRANSCEND) | Double blind randomised trial with placebo control group |
| Yasar et al 2013 | Ginkgo Evaluation of Memory Study (GEMS) | Double blind randomised placebo controlled trial of Ginkgo Biloba supplementation.  Analyses reported are post hoc examination of antihypertensive treatment and risk of Alzheimer's Disease |
| Gelber et al 2013 | Honolulu Asia Aging Study | Cohort study |
| Solfrizzi et al 2013 | Italian Longitudinal Study on Aging (ILSA) | Cohort Study |
| Peters et al 2015 | The Newcastle 85+ Study | Cohort Study |
| Chuang et al 2014 | Cache County study | Cohort Study |
| Li et al 2010 | Study using the Administrative database of the US VeteransAffairs (fiscal year 2002-fiscal year 2006) | Cohort analysis |
| Hsu et al 2013 | Study using the Taiwan National Health Insurance database 2000-2006 | Cohort analysis |
| Johnson et al 2012 | Study using a cohort drawn from the Veterans Administration in and outpatient records database | Cohort analysis |
| Tully et al 2016 | Meta-analysis reporting unpublished data from the 90+ Study | Cohort reported within a meta-analysis |
| Tully et al 2016 | Meta-analysis reporting unpublished data from the Three Cities Study | Cohort reported within a meta-analysis |
| Davies et al 2014 | Study using the UK General Practice Research Database | Case control analysis |
| Wagner et al 2012 | Study using data from the Disease Analyser Database (IMS Health Germany). | Case control analysis |
