# Supplementary material for: Relationship Between Antihypertensive Medications and Cognitive Impairment: Part I. Review of Human Studies and Clinical Trials
Source: Curr Hypertens Rep. 2016 Aug 5;18:67. doi: 10.1007/s11906-016-0674-1 (PMC4975763; doi:10.1007/s11906-016-0674-1)
Supplement: Supplementary file 3 — (DOCX 21.7 kb) [file 11906_2016_674_MOESM3_ESM.docx]

Table B Antihypertensive medication classes reported by each human study at baseline

| Author  . . | Study name | Baseline % taking CCB | Baseline % taking ACE | Baseline % taking ARB | Baseline % taking BB | Baseline % taking diuretics | Baseline % taking thiazide like diuretics |
| --- | --- | --- | --- | --- | --- | --- | --- |
| Anderson et al 2011 | Data from the Ongoing Telmisartin Alone and in Combination with Ramipril Global Endpoint Trial (ONTARGET) | Patients randomised 1:1:1 to ARB, ACE I or both | | | | | |
| Anderson et al 2011 | Data from the Telmisartin Randomised Assessment Study in ACE Intolerant Subjects with Cardiovascular Disease trial (TRANSCEND) | Patients randomised 1:1 to ARB or placebo | | | | | |
| Yasar et al 2013 | Ginkgo Evaluation of Memory Study (GEMS) | 333 (14.8%) | 324(14.4%) | 140(6.2%) | 457(20.3%) | 351(15.6%) | Not reported |
| Gelber et al 2013 | Honolulu Asia Aging Study | 299(13.6) CCB alone | 100(4.6) ACI alone | Not reported | 153(7.0) BB alone | 153(7.0) diuretics alone | Not reported |
| Solfrizzi et al 2013 | Italian Longitudinal Study on Aging (ILSA) | Exposed to antihypertensive not including ACE-I 33.4%  Exposed to ACE 23.4% | | | | | |
| Peters et al 2015 | The Newcastle 85+ Study | 31% | 25% | 13.90% | 32.80% | Thiazide and related diuretics. 25.2% |  |
| Chuang et al 2014 | Cache County study | At some point during follow up, | | | | | |
|  |  | 670 (33.6%) CCBs,  (47.3% dihydropiridine, 52.7% nondihydropiridine) | 801 (40.2%) | Not reported | 717 (33.6%) | 1253 (62.9%) | 75% of all diuretics were thiazides |
| Li et al 2010 | Study using the Administrative database of the US Veterans Affairs (fiscal year 2002-fiscal year 2006) | Not reported | AD cohort; lisinopril 93484 (11.4) Dementia cohort; lisinopril91164 (11.4) | AD cohort 11703 (1.4). Dementia cohort 11507 (1.4). | Not reported | Not reported | Not reported |
| Author | Study name | Baseline % taking CCB | Baseline % taking ACE | Baseline % taking ARB | Baseline % taking BB | Baseline % taking diuretics | Baseline % taking thiazide like diuretics |
| Hsu et al 2013 | Study using the Taiwan National Health Insurance database 2000-2006 | ARB group 83.48%,  non-ARB group 85.5% | Not reported | ARB group were 50% of the total sample - see Source of subjects | ARB group 72.22%,  non-ARB group 72.49% | Not reported | ARB group 19.67%,  non-ARB group 28.29% |
| Johnson et al 2012 | Study using a cohort drawn from the Veterans Administration in and outpatient records database | 31.90% | 53% | 4.20% | 30.50% | 40.80% | Not reported |
| Tully et al 2016 | Meta-analysis reporting unpublished data from the 90+ Study | Not reported | Not reported | Not reported | Not reported | Exposure of interest in the meta-analysis was diuretic use. Details of % taking diuretics in the constituent studies not reported. | Not reported |
| Tully et al 2016 | Meta-analysis reporting unpublished data from the Three Cities Study | Not reported | Not reported | Not reported | Not reported |  | Not reported |
| Davies et al 2014 | Study using the UK General Practice Research Database | Cases 42%, controls 45% | Cases 35%, controls 38% | Cases 5%, controls 7% | Cases 41%, controls 42% | Not reported | Cases 50%, controls 53% |
| Wagner et al 2012 | Study using data from the Disease Analyser Database (IMS Health Germany). | Cases 5.3%; Controls 5.8% | Cases 5.8%; Controls 4.4% | Cases 8.9%; Controls 10.6% | Cases 4.7%; Controls 6.8% | Cases 1.2%; Controls 1.4% | Not reported |
