# Supplementary material for: Relationship Between Antihypertensive Medications and Cognitive Impairment: Part I. Review of Human Studies and Clinical Trials
Source: Curr Hypertens Rep. 2016 Aug 5;18:67. doi: 10.1007/s11906-016-0674-1 (PMC4975763; doi:10.1007/s11906-016-0674-1)
Supplement: Supplementary file 4 — (DOCX 29.7 kb) [file 11906_2016_674_MOESM4_ESM.docx]

Table C1 Quality Assessment Human Studies: Recruitment bias

| Author | Study name | Clearly focused issue | Appropriate method to answer q | Recruitment bias |
| --- | --- | --- | --- | --- |
| Anderson et al 2011 | Data from the Ongoing Telmisartin Alone and in Combination with Ramipril Global Endpoint Trial (ONTARGET) | Yes | Yes | Some. Participants were required to meet trial inclusion criteria - not a population sample |
| Anderson et al 2011 | Data from the Telmisartin Randomised Assessment Study in ACE Intolerant Subjects with Cardiovascular Disease trial (TRANSCEND) | Yes | Yes | Some. Participants were required to meet trial inclusion criteria - not a population sample |
| Yasar et al 2013 | Ginkgo Evaluation of Memory Study (GEMS) | Yes - secondary analysis of trial data | Yes not a trial, however, use of cohort study appropriate | Yes. Likely to be a selected group as drawn from a trial population with follow up and without concomitant antihypertensive medication |
| Gelber et al 2013 | Honolulu Asia Aging Study | Yes | Yes not a trial, however, use of cohort study appropriate | Yes - only Japanese American men living in Oahu and participating in an existing study |
| Solfrizzi et al 2013 | Italian Longitudinal Study on Aging (ILSA) | Yes | Yes not a trial, however, use of cohort study appropriate | Yes, a subset of a population sample |
| Peters et al 2015 | The Newcastle 85+ Study | Yes | Yes not a trial, however, use of cohort study appropriate | Yes, a subset of a population sample but similar profile to whole baseline cohort |
| Chuang et al 2014 | Cache County study | Yes | Yes not a trial, however, use of cohort study appropriate | No. Population selection and 90% consented. Appropriate sample. |
| Li et al 2010 | Study using the Administrative database of the US Veterans Affairs (fiscal year 2002-fiscal year 2006) | Yes | Some. Retrospective analysis of a cohort from an existing database has limitations; however, it did include large numbers. | Yes only those with data in the database and taking the selected medications. Participant population also largely male. |
| Hsu et al 2013 | Study using the Taiwan National Health Insurance database 2000-2006 | Yes | Some. Retrospective analysis of a cohort from an existing database has limitations; however, it did include large numbers. | Yes only those with data in the database defined as cases or controls and taking the selected medications although authors comment that the parent database is representative of the Taiwan population. |
| Johnson et al 2012 | Study using a cohort drawn from the Veterans Administration in and outpatient records database | Yes | Retrospective analysis of a cohort from an existing database has limitations; however, it did include large numbers. | Yes only those with data in the database and with diabetes |
| Tully et al 2016 | Meta-analysis reporting unpublished data from the 90+ Study | Yes | Yes but limited information available as study results are unpublished data from a meta-analysis | Unclear as study results are unpublished data from a meta-analysis |
| Tully et al 2016 | Meta-analysis reporting unpublished data from the Three Cities Study | Yes | Yes but limited information available as study results are unpublished data from a meta-analysis | Unclear as study results are unpublished data from a meta-analysis |
| Davies et al 2014 | Study using the UK General Practice Research Database | Yes | Retrospective case control analysis from an existing database has limitations; however, it did include large numbers. | Yes only those with data in the database and taking the selected medications and potential for prescription bias although authors comment that the parent database is representative of the UK population. |
| Wagner et al 2012 | Study using data from the Disease Analyser Database (IMS Health Germany). | Yes | Some. Retrospective case control analysis from an existing database has limitations. | Yes only those in the database cases and matched control with at least one consultation per year during the study period. |

Table C2 Quality Assessment Human Studies: Bias in exposure and outcome

| Author | Exposure bias (intervention) | Bias in outcome measurement | Bias in assessment of confounders |
| --- | --- | --- | --- |
| Hsu et al 2013 | Possible bias. Exposure to ARBs required prescription of a minimum of 90 pills during the observation period. Unclear beyond this point. Possible that prescription influenced by factors that also influenced cognitive function. | Possible bias. Potential for under reporting as lack of coded diagnoses may not represent lack of disease and diagnostic procedures may not have been equitable across all medical environments reporting onto the database. | Possible bias. Core confounders were included; however, lack of adjustment for blood pressure is a limitation. Under reporting of confounders may also be present as lack of a disease or symptom code may not indicate lack of the disease / symptom. |
| Johnson et al 2012 | Drug exposure stated as the period prevalence of any antihypertensive class during the calendar year 2000 with data from prescribing records. Drug classes defined as present or absent. Long term exposure not assessed. | Potential for under reporting as lack of coded diagnoses may not represent lack of disease and diagnostic procedures may not have been equitable across all medical environments reporting onto the database. | Unclear as details not provided. |
| Tully et al 2016 | Unclear as study results are unpublished data from a meta-analysis | Unclear as study results are unpublished data from a meta-analysis | Unclear as study results are unpublished data from a meta-analysis |
| Tully et al 2016 | Unclear as study results are unpublished data from a meta-analysis | Unclear as study results are unpublished data from a meta-analysis | Unclear as study results are unpublished data from a meta-analysis |
| Davies et al 2014 | Minimum exposure required. Possible that prescription influenced by factors that also influenced cognitive function although use of up to 8 years’ time lag makes this less likely. | Potential for under reporting as lack of coded diagnoses may not represent lack of disease and diagnostic procedures may not have been equitable across all medical environments reporting onto the database. | Core confounders were included. Under reporting of confounders may also be present as lack of a disease or symptom code may not indicate lack of the disease / symptom. |
| Wagner et al 2012 | Yes - length of exposure unclear and potential for prescription bias | Possible bias. Potential for under reporting as lack of coded diagnoses may not represent lack of disease and diagnostic procedures may not have been equitable across all medical environments reporting onto the database. | Possible bias. Core confounders were included. Under reporting of confounders may also be present as lack of a disease or symptom code may not indicate lack of the disease / symptom. |

| Li et al 2010 | Possible bias. Exposure to selected treatments at minimum of 80% of first 6 months in the study, unclear beyond this point. Additional assessment of those who switched drug from ARB to ACE-I or back also assessed. Possible that prescription influenced by factors that also influenced cognitive function. | Possible bias. Potential for under reporting as lack of coded diagnoses may not represent lack of disease and diagnostic procedures may not have been equitable across all medical environments reporting onto the database. | Possible bias. Core confounders were included, however, lack of adjustment for blood pressure is a limitation and authors comment that other confounders may be present but data not available e.g. education. Under reporting of confounders may also be present as lack of a disease or symptom code may not indicate lack of the disease / symptom. |
| --- | --- | --- | --- |

| Author  Table C3 Quality Assessment Human Studies: Bias in follow-up | Bias in follow up - patient loss | Bias in follow up - length | Randomisation method | Concealment of allocation | Blinding allocation | Blinding of outcome assessment | Attrition bias | Selective outcome reporting bias |
| --- | --- | --- | --- | --- | --- | --- | --- | --- |
| Anderson et al 2011 | Information of loss to follow up provided - some differences across randomised groups. | Unclear as detailed follow up information not reported. | Adequate | Blinded with matching control (double dummy) | Blinded | Blinded | Information of loss to follow up provided - some differences across randomised groups. | Outcome identified following use of standard screening tool at study visits regardless of randomised group |
| Anderson et al 2011 | Information of loss to follow up provided - some differences across randomised groups. | Unclear as detailed follow up information not reported | Adequate | Blinded with matching placebo | Blinded | Blinded | Information of loss to follow up provided - some differences across randomised groups. | Outcome identified following use of standard screening tool at study visits regardless of randomised group |
| Yasar et al 2013 | Unknown - only those with follow up were included | No, follow up sufficient to identify incident cognitive impairment | N/A not a clinical trial | N/A not a clinical trial | N/A not a clinical trial | Not reported | See previous | None |
| Gelber et al 2013 | Some. Analysis based on cohort with follow up data available | No, follow up sufficient to identify incident dementia | N/A not a clinical trial | N/A not a clinical trial | N/A not a clinical trial | Not reported | See previous | None |
| Solfrizzi et al 2013 | Yes, participants lost for a variety of reasons. | No | N/A not a clinical trial | N/A not a clinical trial | N/A not a clinical trial | Not clear whether outcome assessment blinded | Yes possible that impaired subjects more likely to drop out or refuse further assessment | No all participants included in analysis seem likely to have had follow up assessment |
| Author | Bias in follow up - patient loss | Bias in follow up - length | Randomisation method | Concealment of allocation | Blinding allocation | Blinding of outcome assessment | Attrition bias | Selective outcome reporting bias |
| Peters et al 2015 | Yes - selected group. Additional analyses in the wider cohort examining antihypertensive class and mortality during the study follow up found no relationships. | No | N/A not a clinical trial | N/A not a clinical trial | N/A not a clinical trials | None, however, assessors collecting data had no role in the analysis. | Yes analyses suggested that impaired subjects more likely to drop out or refuse further assessment | No all participants included in analysis seem likely to have had follow up assessment |
| Chuang et al 2014 | Possible bias, reasons and timing of loss not clear | Follow up length long enough to assess incident dementia. Details of mean follow up and reasons for withdrawal not reported. | N/A not a clinical trial | N/A not a clinical trial | N/A not a clinical trial | Not clear whether outcome assessment blinded | Yes possible that impaired subjects more likely to drop out or refuse further assessment | None |
| Li et al 2010 | Possible bias. All participants were censored, however, numbers lost to follow up are unclear and there is the possibility that some participants may effectively have been lost to follow up due to lack of engagement with medical professionals over the study period. | Follow up of sufficient length to assess incident dementia. Lost to follow up numbers and reasons not clear. | N/A not a clinical trial | N/A not a clinical trial | N/A not a clinical trial | No. Outcome as reported in the Veterans affairs database, diagnoses not influenced by the study. | Possible bias, numbers lost to follow up/death or potential loss of follow up or lack of follow up assessment unclear. | Possible bias. Potential for under reporting as lack of coded diagnoses may not represent lack of disease. |
| Author | Bias in follow up - patient loss | Bias in follow up - length | Randomisation method | Concealment of allocation | Blinding allocation | Blinding of outcome assessment | Attrition bias | Selective outcome reporting bias |
| Hsu et al 2013 | Possible bias. Details not reported although likely to be low as the data source covers the majority of the population and there is the possibility that some participants may effectively have been lost to follow up due to lack of engagement with medical professionals over the study period. | Mean follow up sufficient to assess incident dementia however, mean duration of time from exposure to outcome not reported. | N/A not a clinical trial | N/A not a clinical trial | N/A not a clinical trial | No. Outcome as reported in the Taiwan National Health Insurance database, diagnoses not influenced by the study. | Possible bias, numbers lost to follow up/death or potential loss of follow up or lack of follow up assessment unclear. | Possible bias. Potential for under reporting as lack of coded diagnoses may not represent lack of disease. |
| Johnson et al 2012 | Unlikely given follow up length but no details given. | 2 years is fairly short to assess the impact of prior treatment on incident dementia. | N/A not a clinical trial | N/A not a clinical trial | N/A not a clinical trial | Outcome as reported in the UK General Practice Research Datalink database, diagnoses not influenced by the study. | Unlikely given follow up length but no details given. | Potential for under reporting as lack of coded diagnoses may not represent lack of disease. |
| Tully et al 2016 | Unclear as study results are unpublished data from a meta-analysis | Unclear as study results are unpublished data from a meta-analysis | N/A not a clinical trial | N/A not a clinical trial | N/A not a clinical trial | Unclear as study results are unpublished data from a meta-analysis | Unclear as study results are unpublished data from a meta-analysis | Unclear as study results are unpublished data from a meta-analysis |
| Author | Bias in follow up - patient loss | Bias in follow up - length | Randomisation method | Concealment of allocation | Blinding allocation | Blinding of outcome assessment | Attrition bias | Selective outcome reporting bias |
| Tully et al 2016 | Unclear as study results are unpublished data from a meta-analysis | Unclear as study results are unpublished data from a meta-analysis | N/A not a clinical trials | N/A not a clinical trials | N/A not a clinical trials | Unclear as study results are unpublished data from a meta-analysis | Unclear as study results are unpublished data from a meta-analysis | Unclear as study results are unpublished data from a meta-analysis |
| Davies et al 2014 | Case control analysis so potential for bias, not clear how representative case control selection was of entire Disease Analyser dataset. | Overall duration of assessment and mean time between exposure and outcome not clear, however, analyses using time lags of up to 8 years are sufficient to allow development of dementia. | N/A not a clinical trial | N/A not a clinical trial | N/A not a clinical trial | Outcome as reported in the GPRD database, diagnoses not influenced by the study. | Possible bias, case control design so selected population. | Potential for under reporting as lack of coded diagnoses may not represent lack of disease. |
| Wagner et al 2012 | Possible bias. Case control analysis so potential for bias, not clear how representative case control selection was of entire GPRD dataset. | Minimum exposure time 3 years, however follow up details not clear | N/A not a clinical trial | N/A not a clinical trial | N/A not a clinical trial | No. Outcome as reported in the Disease Analyser affairs database, diagnoses not influenced by the study. | Possible bias, case control design so selected population. | Possible bias. Potential for under reporting as lack of coded diagnoses may not represent lack of disease. |
